# Removing barriers to sodium reduction: Focusing on practice

**DOI:** 10.1111/jch.14343

**Published:** 2021-08-03

**Authors:** Yu Yan, Jianjun Mu

**Affiliations:** ^1^ Department of Cardiovascular Medicine First Affiliated Hospital of Medical College Xi'an Jiaotong University and Key Laboratory of Environment and Genes Related to Diseases Ministry of Education Xi'an People's Republic of China

Hypertension, as the most common preventable risk factor for cardiovascular disease and all‐cause mortality, has become a public health burden worldwide.^1^ It is estimated that 1.13 billion people globally have hypertension, but fewer than one in five hypertensive patients have the problem under control.^2^ Among many established risk factors for hypertension, excessive sodium intake is of great concern.^3^ Numerous evidence from animal studies, epidemiological studies, clinical trials, and meta‐analyses of trials have demonstrated that dietary sodium reduction is associated with decreased blood pressure and reduced risk of cardiovascular disease and all‐cause mortality.^4^ In fact, reducing dietary sodium intake has been recommended in hypertension guidelines and population dietary policies in many countries.^5^ Although considerable effort has been made in reducing salt intake since 2013, the current global salt consumption levels of 9–12 g/day is still far exceeding 5 g/day, recommended by the World Health Organization (WHO).^6^, ^7^

In response to the unsatisfying results of recent attempts in dietary sodium reduction, a group of Thai researchers, Sirichai and colleagues, developed a user‐friendly salt meter device to monitor sodium intake.^8^ A randomized controlled trial is conducted among uncontrolled hypertensive patients to evaluate the efficacy of using salt‐meter combined with dietary education in reducing dietary salt consumption. The device uses the alternating electrical current to determine the concentration of sodium chloride in food (such as curry, soup, or broth), which is displayed as corresponding emotional faces on the screen. The study shows that compared with only dietary education, additional implementation of salt meter performs better in reducing 24‐h urine sodium excretion and blood pressure in uncontrolled hypertensive patients after the 8‐week intervention. More importantly, during the follow‐up period, all patients who received the salt meters were in good compliance in usage. They concluded that the salt meter would help in dietary salt intake reduction, and therefore blood pressure management in uncontrolled hypertensive patients in real life.

General broad recommendations on sodium reduction have been considered to be among the most cost‐effective strategies for promoting general population health outcomes. Improvements of knowledge, attitudes, and practices (KAP) on salt intakes are considered as the key components of effective sodium reduction strategies. Among the three components, improvement of knowledge is fundamental and essential for stimulating a positive attitude towards sodium reduction, which would influence the behaviors of the individuals and the groups, determining the efficiency of sodium reduction strategies. The most recommended sodium‐related behaviors include the use of scaled salt spoons, the evaluation of sodium content in processed food, and the implementation of educational campaigns. However, shifting the dietary habit in high salt‐intake population is not easy and requires multidimensional approaches. Previous observational studies showed that despite good levels of knowledge and favorable attitudes towards sodium reduction, only a small proportion of subjects had taken action to reduce salt intake (Table 1).^9^, ^10^, ^11^, ^12^, ^13^, ^14^ Complementary approaches are needed to remove the barrier to salt reduction and increase the accessibility of a low‐sodium lifestyle. The new salt meter device developed by Sirichai and colleagues was a commendable example of assisting in dietary sodium reduction. The device provides a tool to help people to be more salt‐conscious and to choose healthier alternatives in a visual manner; it makes complex measures more accessible, apprehensible, and applicable. Such initiatives contribute to lowering sodium intake as a practice component of KAP.

**TABLE 1:** Recent evidence regarding knowledge, attitudes, and practice related to salt reduction

First author, year, country	Study design	Population description	No. of participants	Major findings
Juan Zhang, 2013, China^9^	cross‐sectional study	aged 18 to 69 years	15,350	Half of the population was aware of the relationship of sodium with hypertension. 70% indicated their intention to reduce sodium intake. Only 39% reported that they had taken action to reduce sodium.
Magali Leyvraz, 2018, Sub‐Saharan African Countries^10^	cross‐sectional study	aged 25 to 65 years	588	85% of the participants knew that high salt intake could cause health problems. 91% of the participants thought it was important to limit salt intake. Only 56% of the respondents often tried to limit their salt intake.
Kamal Ghimire, 2019, Nepal^11^	cross‐sectional study	mean age 45.2 years	2815	86.3% answered that high salt intake would cause health problems. 88.7% reported that it is important to reduce dietary salt. 64.7% of the respondents reported that they rarely or never added extra salt in their food at the table.
Si Chen, 2020, China^12^	cross‐sectional study	consumers from six of seven major geographical regions in China	2430	83.0% of the participants believed that eating less salt would benefit their health. 65.6% of the participants expressed interest in lowering their salt intake. Only a quarter of the participants claimed that they made an ongoing effort to reduce salt.
Mahitab A Hanbazaza, 2020, Saudi Arabia^13^	cross‐sectional study	aged 20 to 50 years	467	The dietary‐salt‐related knowledge, attitude, and habits toward salt reduction were positively correlated; however, knowledge and attitudes were not significantly correlated with consumption.
Pimbucha Rusmevichientong, 2021, Thailand^14^	cross‐sectional study	over the age of 18 years	376	More than 50% of the subjects had knowledge about high salt food consumption leading to higher chances of developing hypertension. Knowledge and attitudes were not significantly correlated with consumption.

Future efforts are expected in the modification of device design to measure solid food for broader application. In addition, the contribution of the salt meter merits further validation in a large, long‐term follow‐up study. Taken together, the salt meter provides new possibilities for sodium reduction practice and may help promote individual dietary change and public sodiumreduction initiatives.

## CONFLICT OF INTEREST

The authors have no competing interests.

## AUTHOR CONTRIBUTIONS

Yu Yan and Jianjun Mu contributed to the conception of this work. Yu Yan wrote the manuscript and Jianjun Mu critically revised it for important intellectual. Both authors approved the final version of the manuscript.

## FUNDING INFORMATION

None.
